# Protein expression in the obligate hydrocarbon‐degrading psychrophile *Oleispira antarctica* RB‐8 during alkane degradation and cold tolerance

**DOI:** 10.1111/1462-2920.14956

**Published:** 2020-02-28

**Authors:** Benjamin H. Gregson, Gergana Metodieva, Metodi V. Metodiev, Peter N. Golyshin, Boyd A. McKew

**Affiliations:** ^1^ School of Life Sciences, University of Essex Colchester, Essex CO4 3SQ UK; ^2^ School of Natural Sciences, College of Environmental Sciences and Engineering, Bangor University Bangor UK; ^3^ Centre for Environmental Biotechnology Bangor University Deiniol Road, Bangor LL57 2UW UK

## Abstract

In cold marine environments, the obligate hydrocarbon‐degrading psychrophile *Oleispira antarctica* RB‐8, which utilizes aliphatic alkanes almost exclusively as substrates, dominates microbial communities following oil spills. In this study, LC–MS/MS shotgun proteomics was used to identify changes in the proteome induced during growth on *n*‐alkanes and in cold temperatures. Specifically, proteins with significantly higher relative abundance during growth on tetradecane (*n*‐C_14_) at 16°C and 4°C have been quantified. During growth on *n*‐C_14_, *O*. *antarctica* expressed a complete pathway for the terminal oxidation of *n*‐alkanes including two alkane monooxygenases, two alcohol dehydrogenases, two aldehyde dehydrogenases, a fatty‐acid‐CoA ligase, a fatty acid desaturase and associated oxidoreductases. Increased biosynthesis of these proteins ranged from 3‐ to 21‐fold compared with growth on a non‐hydrocarbon control. This study also highlights mechanisms *O*. *antarctica* may utilize to provide it with ecological competitiveness at low temperatures. This was evidenced by an increase in spectral counts for proteins involved in flagella structure/output to overcome higher viscosity, flagella rotation to accumulate cells and proline metabolism to counteract oxidative stress, during growth at 4°C compared with 16°C. Such species‐specific understanding of the physiology during hydrocarbon degradation can be important for parameterizing models that predict the fate of marine oil spills.

## Introduction

*Oleispira antarctica* RB‐8 is a psychrophilic aerobic bacterium belonging to the *Gammaproteobacteria* class, which was first isolated from oil‐enriched microcosms containing seawater from Rod Bay (Ross Sea, Southern Antarctica) (Yakimov *et al*., 2003). *Oleispira antarctica* is a member of a group of organisms known as obligate hydrocarbonoclastic bacteria (OHCB) which grow on a highly restricted spectrum of substrates, predominantly aliphatic hydrocarbons. Oil pollution in marine environments rapidly induces a bloom of OHCB which can constitute up to 80%–90% of the microbial community (Harayama *et al*., 1999; Kasai *et al*., 2002). *Oleispira* shares many traits with other described genera of marine OHCB, such as *Alcanivorax* (Yakimov *et al*., 1998) and *Thalassolituus* (Yakimov *et al*., 2004) including their marine origin, purely respiratory metabolism and the capability of growth almost exclusively on aliphatic alkanes and their derivatives. However, in contrast with these genera, which contain species that are characterized by mesophilic behaviour, *O*. *antarctica* has a broad growth temperature optimum between 1°C and 15°C (Yakimov *et al*., 2003). The minimal growth temperature is estimated to be −6.8°C using the Ratkowsky square root temperature growth model (Ratkowsky *et al*., 1983). Around 90% of the biosphere exists at temperatures below 10°C, and this provides ample opportunity for *Oleispira* spp. to dominate microbial communities due to their ecological competitiveness in cold environments (Feller and Gerday, 2003; Hazen *et al*., 2010; Mason *et al*., 2012; Kube *et al*., 2013).

The GenBank and RDP databases contain 16S rRNA gene sequences of 25 *Oleispira* bacteria originating from microbial communities from cold environments such as Arctic sea ice (Gerdes *et al*., 2005; Brakstad *et al*., 2008), sediments (Dong *et al*., 2015) an epishelf lake (Veillette *et al*., 2011), Antarctic and subantarctic seawater (Prabagaran *et al*., 2007; Singh *et al*., 2015), seawater from the Norwegian fjord Trondheimsfjord (Brakstad and Bonaunet, 2006) and the Irish and North Seas (Coulon *et al*., 2007; Gertler *et al*., 2012). Warmer locations include fish farm sediments in Southern Tasmania (Bissett *et al*., 2006), marine basalts from the East Pacific Rise (Mason *et al*., 2007) and coastal seawater (Wang *et al*., 2012).

Previous reports have shown that *Oleispira* species can be among the most dominant community members in the presence of aliphatic petroleum hydrocarbons at lower temperatures and can outcompete other OHCB such as *Thalassolituus* (Coulon *et al*., 2007), or *Alcanivorax*, which is often the most dominant alkane degrader in marine oil spills (Harayama *et al*., 2004; Kostka *et al*., 2011). For example, bacteria related to *O*. *antarctica* (98% 16S rRNA similarity) became the most dominant bacteria in crude‐oil amended North Sea Thames Estuary microcosms incubated at 4°C, in contrast to microcosms incubated at 20°C where the abundance of *Thalassolituus* was highest (Coulon *et al*., 2007). *Oleispira* were also found to be predominant in cold deep‐water samples (depth: 1099–1219 m, average temperature 4.7°C) after the Deepwater Horizon oil spill in the Gulf of Mexico (Hazen *et al*., 2010; Mason *et al*., 2012). Furthermore, *Oleispira* sequences were present at a high abundance (14%–17% of recovered reads) in 16S rRNA gene libraries of oil‐enriched microcosms from the Gulf of Mexico grown at 5°C (Techtmann *et al*., 2017).

The genome sequencing of *O*. *antarctica* RB‐8 revealed an array of genes putatively involved in adaption to cold environments such as chaperonins, Cpn60 (C29610) and Cpn10 (C29620), which can reduce the maximum growth temperature of mesophiles (Ferrer *et al*., 2003; Kube *et al*., 2013). Analysis of the chaperonin interactome suggests that protein‐chaperone interactions are protecting functionality of proteins at low temperatures (Strocchi *et al*., 2006; Kube *et al*., 2013). The genome sequencing also revealed putative genes for hydrocarbon degradation including three alkane monooxygenase and one P450 cytochrome gene. The first alkane monooxygenase gene (C23040) has its closest homologue in AlkB2 from *A*. *borkumensis* SK2 and is also encoded next to the transcriptional regulator GntR. Two other alkane monooxygenases (C34350 and C34450) are clustered within putative operons (C34330‐C34360 and C34430‐C4450 respectively) both with genes that code for a transcriptional regulator of the AraC family and an oxidoreductase. The first operon also contains an alcohol dehydrogenase (C34360) which is similar to the alcohol dehydrogenase AlkJ from *A*. *borkumensis* SK2. Greater mRNA expression of these genes in *O*. *antarctica* was quantified during growth on tetradecane (*n*‐C_14_) (compared with the non‐hydrocarbon substrate acetate) through targeted quantitative reverse transcription polymerase chain reaction (Kube *et al*., 2013). However, mRNA abundance was only calculated for three *alkB* genes and one *P450* gene. To date, their translation has not been confirmed at the protein level and no study has compared expression of either a total transcriptome or shotgun proteome in *O*. *antarctica* during growth on alkanes versus a non‐hydrocarbon control or at different temperatures. Comparing protein expression during growth on a petroleum hydrocarbon versus a non‐hydrocarbon substrate will enable us to see the compositional and abundance changes within the total proteome to identify the key enzymes involved in the metabolism of these crude oil components. By measuring the proteomic response at different temperatures, we can also understand potential mechanisms *O*. *antarctica* uses which provides it ecological competitiveness over other hydrocarbon‐degrading bacteria and why it regularly dominates oil‐contaminated environments at low temperatures. Such bottom‐up experimental approaches that yield a species‐specific understanding of hydrocarbon degradation can be important for parameterizing future improved models that predict the biodegradative fate of marine oil spills by complex microbial communities (Röling and van Bodegom, 2014). There is a growing interest in post‐spill monitoring of microbial communities (Kirby *et al*., 2018), and an understanding of the key proteins involved in hydrocarbon degradation is important for designing gene primers that can be used in monitoring tools that are based on the abundance of specific functional genes. This can complement current tools based on community 16S rRNA genes, such as the Ecological Index of Hydrocarbon Exposure (Lozada *et al*., 2014).

In this study, we used LC–MS/MS shotgun proteomics to identify changes in the proteome induced during growth on alkanes and at cold temperatures. Specifically, proteins with significant increases in spectral counts during growth on *n*‐C_14_ at 16°C and 4°C have been quantified, giving a unique insight in the proteins involved in the degradation of alkanes and cold adaptation.

## Results

### *Growth on alkanes*

Preliminary growth tests to determine the alkane degradation range of *O*. *antarctica* RB‐8 were performed with alkanes in the size range from 10 carbons atoms (decane) to 32 carbon atoms (dotriacontane) including *n*‐C_10_, *n*‐C_12_, *n*‐C_16_, *n*‐C_20_, *n*‐C_24_, *n*‐C_28_ and *n*‐C_32_ and the branched alkane pristane (2,6,10,14‐tetramethylpentadecane) with the degradation of these compounds confirmed and quantified through GC–MS analysis. *Oleispira antarctica* RB‐8 grew rapidly at both 4°C or 16°C on *n*‐alkanes from *n*‐C_10_ to *n*‐C_24_ after a 3‐day lag phase but was unable to grow on the larger *n*‐alkanes or on the branched alkane pristane (no growth was observed after 21‐day incubation) (Fig. S1). There was no significant difference of growth rates between substrates or temperature (Table S1). End point GC–MS analysis revealed significant (*P* < 0.05) alkane degradation in all single substrate microcosms up to *n*‐C_24_ after 21 days ranging from 42% to 81% with no significant differences after growth at 4°C or 16°C (Fig. S2). Differences in the proteomes were quantified cells growing at either 4°C or 16°C on the medium‐chain *n*‐alkane tetradecane (*n*‐C_14_), which *O*. *antarctica* was originally isolated on (Yakimov *et al*., 2003), versus cells growing on the non‐hydrocarbon control Tween 80 (one of the very few non‐hydrocarbon substrates OHCB can utilize).

### *Proteomic overview*

**Figure 1:**
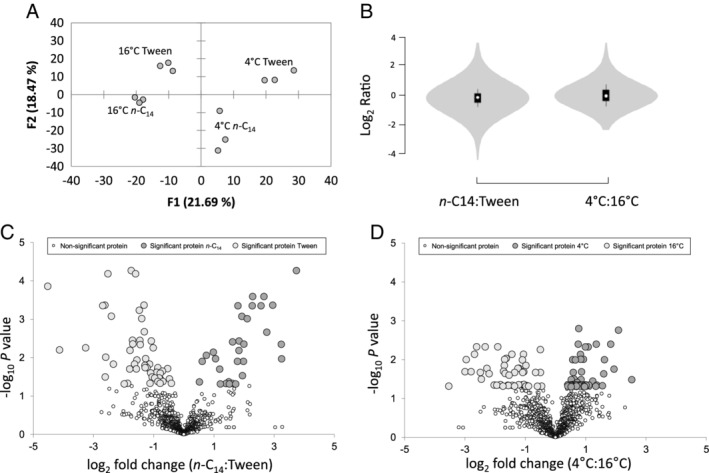
A. Principle component analysis of replicate *Oleispira antarctica* RB‐8 proteomes during growth on tetradecane (*n*‐C_14_) and the non‐hydrocarbon control Tween 80 (Tween) at 4°C and 16°C based on normalized spectral counts for proteins. B. Violin plots of normalized LC–MS/MS spectral counts showing the distribution of detected proteins in *O*. *antarctica* RB‐8 during growth on different substrates; *n*‐C_14_ versus Tween (left; *n*‐C14:Tween); and different temperature; 4°C versus 16°C (right; 4°C: 16°C). C and D. Volcano plots of normalized LC–MS/MS spectral counts comparing *O*. *antarctica* RB‐8 protein abundance during growth on different substrates; *n*‐C_14_ versus Tween (left; *n‐*C_14_: Tween) and different temperatures; 4°C versus 16°C (right; 4°C:16°C). Larger data points (light and dark grey) represent differentially expressed proteins with *P‐*values below 0.05.

A total of 148,648 spectra were assigned to 1246 proteins (average of 12,387 spectral counts per replicate and ranging from 1 to 4772 spectral counts per protein), representing detection of simultaneous expression of ~32% of the total genome that contains 3919 protein coding genes (Table S2). The effect of both growth substrate and temperature led to expression of differing proteomes for each treatment, with highly similar proteomes observed between the biological replicates (Fig. 1A). The vast majority of detected proteins were expressed in highly similar abundance regardless of growth substrate or temperature, with a smaller subset of proteins differing in their expression ratio (Fig. 1B–D). Overall, 286 were significantly differentially expressed across all four treatments (Table S2). A total of 84 proteins were significantly differentially expressed because of the growth substrate, of which 31 proteins showed significant increases in biosynthesis when growing on *n*‐C_14_ (Fig. 1C). A similar amount of proteins (95) were differentially expressed due to temperature (T_opt_: 1°C–15°C), with the relative abundance of 47 significantly increasing at the cold temperature of 4°C (Fig. 1D).

### *Alkane‐induced proteins*

**Figure 2:**
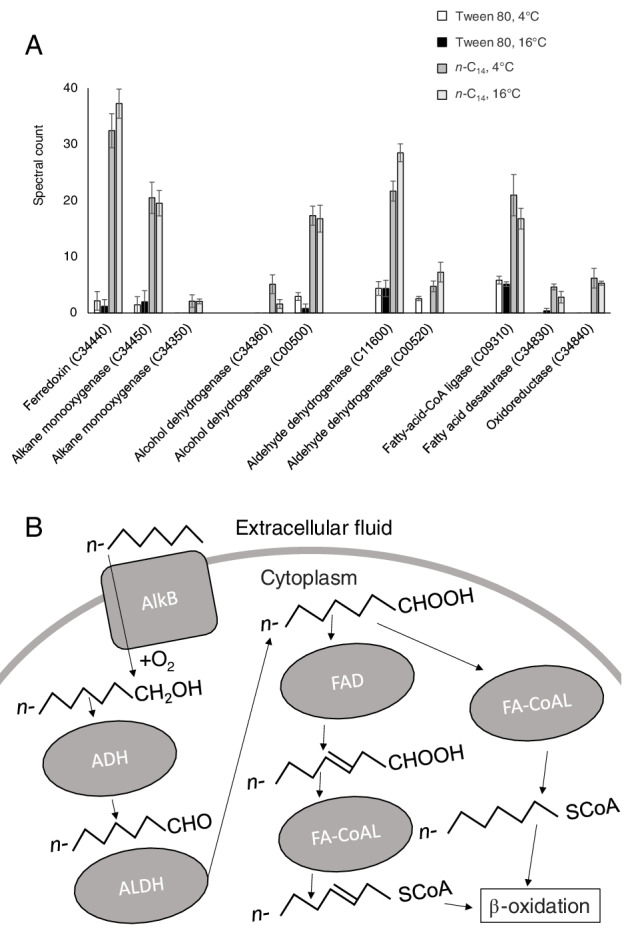
A. Normalized spectral counts (means ± SE; *n* = 3) of differentially expressed alkane degradation proteins during growth on the *n*‐alkane tetradecane (*n*‐C_14_; light grey and dark grey), the non‐hydrocarbon control (Tween 80; white and black) at 4° and 16°C in *Oleispira antarctica* RB‐8. B. The monooxygenase (AlkB; C34350/C34450) introduces oxygen into the *n*‐alkane converting it into a primary alcohol. This alcohol is further oxidized to an aldehyde and then a fatty acid by the alcohol dehydrogenase (ADH; C00500/C34360) and aldehyde dehydrogenase (ALDH; C00520/C11600) respectively. The fatty acid desaturase (FAD; C34830) incorporates double bonds into the hydrocarbon chain of the saturated fatty acid to yield unsaturated fatty acids. The fatty acid‐CoA ligase (FA‐CoAL; C09310) catalyses the conversion of unsaturated or saturated fatty acids to their active form acyl‐CoAs for degradation via β‐oxidation.

Of the proteins with increased biosynthesis during growth on *n*‐C_14_, many were identified by sequence homology as having specific functions in the hydrocarbon degradation pathway (Fig. 2). Two alkane monooxygenases had higher spectral counts during growth on *n‐*C_14_ (UniProt IDs R4YQX3 and R4YQY4; Fig. 2). These proteins are required for the first and rate‐limiting step of alkane degradation, the introduction of an oxygen atom converting the *n*‐alkane into a primary alcohol. The genes coding for these two monooxygenases (C34350 and C34450) are part of gene cassettes (C34330–C34360 and C34430–C34450) which are situated outside the predicted genomic islands (GIs) and contain genes that code for transcriptional regulators of the AraC family (Gallegos *et al*., 1997), oxidoreductases, alkane monooxygenases and an alcohol dehydrogenase. The alkane monooxygenase R4YQX3 (coded by C34350) had increased biosynthesis together with AlkJ an alcohol dehydrogenase R4YRA7, coded by the adjacent gene C34360) (Fig. 2). Another alkane‐1‐monoxygenase (R4YQY4; coded by the gene C34450) was the most abundantly detected alkane monooxygenase, and the spectral count was significantly 12‐fold higher during growth on *n‐*C_14_ along with an oxidoreductase (R4YRS0) coded by the adjacent gene (C34440) which was 21‐fold higher (Fig. 2). Domain analysis revealed a 2Fe‐2S iron–sulfur cluster binding domain, and the protein is a member of the Fer2 (PF00111) family, suggesting it is a ferredoxin.

The AlkJ (R4YRA7/C34360) protein, which was exclusively detected during growth on *n*‐C_14_ and coexpressed with the alkane monooxygenase (R4YQX3/C34350), is required for the second step of alkane degradation, which is the dehydrogenation of the primary alcohol to yield an aldehyde. Another alcohol dehydrogenase (R4YJ92) also showed increased biosynthesis during growth on the *n*‐alkane. This protein, whose spectral count was nine‐fold higher during growth on *n*‐C14, is coded by the gene C00500 that is part of a putative operon which also contains genes that code for an uncharacterized protein with a signal sequence twin‐arginine motif (R4YJC5/C00510), an aldehyde dehydrogenase (R4YMB5/C00520) and a transcriptional regulator (R4YPZ8/C00530).

The third step in the alkane degradation pathway is the oxidation of the aldehyde, and two aldehyde dehydrogenases, coded by genes at separate loci (C00520 and C11600), that could putatively catalyse this reaction, had significantly higher spectral counts (Fig. 2). The first (R4YMB5) was approximately four‐fold more abundant during growth on *n*‐C_14_, contains an aldehyde dehydrogenase activity domain (pfam00171) and is NAD(P)‐dependent. The second aldehyde dehydrogenase (R4YL03) also had approximately a six‐fold higher spectral count during growth on *n*‐C_14_.

The oxidation of the aldehyde yields a complex fatty acid and proteins involved in their catabolism which showed increased biosynthesis included a medium‐chain fatty acid CoA ligase (R4YKT2/C09310; three‐fold higher spectral counts) involved in the activation of the fatty acid for beta oxidation, and a fatty acid desaturase (R4YV65/C34830; four‐fold higher spectral count) (enzymes that incorporate double bonds into the hydrocarbon chains of fatty acids to yield unsaturated fatty acids prior to activation for beta oxidation) and its associated oxidoreductase (R4YRU8/C34840; exclusively expressed on *n*‐C_14_) (Fig. 2).

### *Cold‐induced proteins*

**Figure 3:**
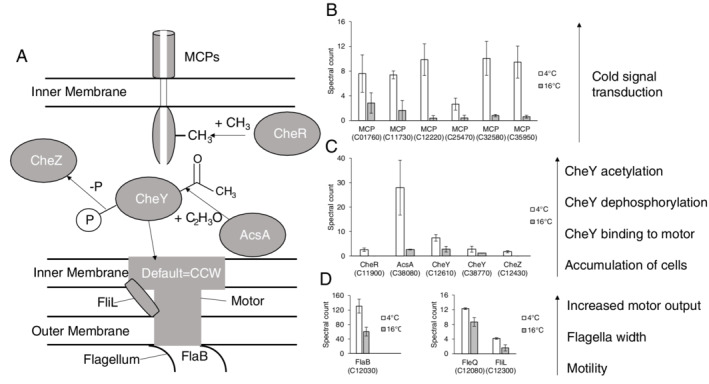
A. Schematic diagram demonstrating the putative roles of proteins with significantly higher spectral counts involved in chemotaxis and motility in *Oleispira antarctica* RB‐8 during growth at 4°C. B–D. Normalized Spectral counts (means ± SE; *n* = 6) for the chemotaxis proteins during growth at 4°C (white) compared with 16°C (grey) in *O*. *antarctica* RB‐8 are presented and separated based on their cellular location. B – Inner membrane; C – Cytoplasmic; D – Outer membrane.

Of the 47 proteins with significantly higher spectral counts during growth at 4°C, 10 (21%) were identified by sequence homology to be involved in chemotaxis and motility (Table S2; Fig. 3A). Six methyl‐accepting chemotaxis proteins (MCPs; R4YJH8/C01760; R4YSY1/C11730; R4YQ71/C12220, R4YT54/C25470, R4YV20/C32580 and R4YRC3/C35950) which are involved in signal transduction to flagella‐associated proteins showed increased biosynthesis with 3‐ to 10‐fold greater spectral counts at 4°C compared with 16°C (Fig. 3B). A methyltransferase, CheR (R4YL24/C11900), which reversibly methylates the MCPs causing clockwise flagella rotation, was exclusively detected at 4°C (Fig. 3C). Methylesterase CheB proteins (R4YJA1; R4YL51; R4YL66) coded by three genes present on the *O*. *antarctica* genome (C00950; C12250; C12450), which reversibly demethylates the MCPs causing anticlockwise flagella rotation, were not detected. Expression of two CheY proteins (R4YLC5/C12610 and R4YUQ1/C38770), which binds to components of the flagella motor also causing clockwise flagella rotation, was 2.6‐fold higher at 4°C (Fig. 3C). An acetyl‐CoA synthetase (AcsA; R4YVD0/C38080) which acetylates the CheY proteins had 12‐fold greater expression at 4°C (Fig. 3B). Flagellin (R4YSZ8/C12030) had 2.2‐fold higher spectral counts at 4°C (Fig. 3D) and based on functional family (FunFam) assignment in CATH, the protein is the B subunit, FlaB (Evalue‐9.4e^−56^). A transcriptional flagella regulator, FleQ (R4YT01/C12080) and the protein FliL (R4YL55/C12300) which contributes to torque generation in flagella at higher motor loads also had 1.4‐fold and 2.5‐fold higher spectral counts respectively at 4°C (Fig. 3D).

**Figure 4:**
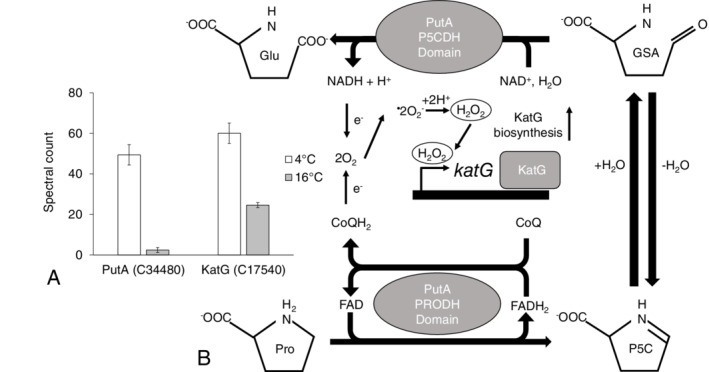
A. Normalized spectral counts (means ± SE; *n* = 6) for the proline utilization A (PutA) flavoenzyme (R4YV58/C34480) and the catalase‐peroxidase KatG (R4YMH2/C17540) with increased biosynthesis during growth at 4°C (white) compared with 16°C (grey) in *Oleispira* RB‐8. B. The proline utilization A (PutA) flavoenzyme consists of a proline dehydrogenase (PRODH) and a Δ1‐pyrroline‐5‐carboxylate dehydrogenase (P5CDH) domains. The PRODH domain contains a flavin adenine dinucleotide (FAD) cofactor and couples the oxidation of proline (Pro) to Δ1‐pyrroline‐5‐carboxylate (P5C) with the reduction of ubiquinone (CoQ). P5C is then hydrolysed to glutamate‐γ‐semialdehyde (GSA) which is oxidized to glutamate (Glu) by the NAD+‐dependent P5CDH domain. Molecular oxygen (O_2_) is reduced by the influx of electrons from electron donors (CoQH2 and NADH) which leads to the formation of superoxide (^•^O_2_). This superoxide is then converted to hydrogen peroxide (H_2_O_2_) either non‐enzymatically or enzymatically by superoxide dismutase. H_2_O_2_ then induces the biosynthesis of the catalase‐peroxidase, KatG, which is active against ROS.

Other proteins with a significant increase in spectral counts at 4°C (Table S2) had roles in proline metabolism, oxidative stress response, RNA metabolism, ribosome maturation and cold tolerance. The spectral counts for the bifunctional protein PutA (R4YV58/C34480) were 26‐fold higher (Fig. 4). For the catalase‐peroxidase KatG (R4YMH2/C17540), they were 2.5‐fold higher. The RNA helicase DeaD (R4YKT5/C10600) was 1.5‐fold greater at 4°C and the ribosome assembly factor, RhlE (R4YQ17/C00680) was 2‐fold higher. Surprisingly, the spectral counts for cold‐adapted chaperonin Cpn60 (R4YPX4/C29610) were 1.5‐fold less abundant at 4°C (Table S2).

## Discussion

### *Growth on alkanes*

Previous studies have not shown the substrate range of *O*. *antarctica* with it only being tested in single substrate microcosms enriched with the *n*‐alkane tetradecane (*n*‐C_14_) (Yakimov *et al*., 2003; Kube *et al*., 2013). *Oleispira antarctica* was unable to grow on long‐chain alkanes greater than 24 carbon atoms long, and this was confirmed by growth tests (Fig. S1). *Oleispira antarctica* may not be able to utilize long‐chain alkanes as it lacks the genes which code for enzymes necessary for degradation (e.g. *almA*, *ladA*), and the low water solubility of these compounds leads to reduced bioavailability (Feng *et al*., 2007; Throne‐Holst *et al*., 2007).

At low temperatures, the growth rate or the rate of substrate oxidation for hydrocarbon‐degrading bacteria is generally observed to decrease, which is thought to be a result of reduced enzymatic activity rates (Bisht *et al*., 2015), but *O*. *antarctica* can maintain rapid growth. Eleven enzymes were cloned and characterized from *O*. *antarctica* and exhibited significant catalytic activity at 4°C but were not truly psychrophilic as they did not show poor activity at higher temperatures (Kube *et al*., 2013). In fact, the enzymes tested had their activity optima at temperatures significantly higher than those in the environment (20°C–50°C). The genome of *O*. *antarctica* revealed that it has acquired a large proportion of its genes, including some of those involved in hydrocarbon metabolism, through horizontal gene transfer (HGT) (Kube *et al*., 2013). There is a possibility these genes were gained from other bacteria with different temperature preferences. Considering that waters in Polar regions hardly warm up above 4°C–6°C and the fact the proteins exhibit generally higher temperature optima indicates some of the enzymes found in *O*. *antarctica* are functioning at a large range of temperatures which is sufficient to facilitate active growth of this bacterium if hydrocarbons become available. In addition, *O*. *antarctica* has been shown to contain chaperonins Cpn60 and Cpn10 that, when expressed heterogeneously, can significantly decrease the growth temperature of a mesophilic host due to their hyperactivation at low temperatures (4°C–12°C) (Ferrer *et al*., 2003). Kube *et al*. (2013) suggests this chaperonin system may fine‐tune central metabolism at low temperatures and indirectly alkane utilization. The origin of the hydrocarbon degradation genes from potentially more mesophilic hydrocarbon degraders, their versatile activity temperature range and the use of a cold‐adapted chaperonin system may explain why there was no significant difference in the growth rates at 4°C and 16°C. The growth rates calculated in this study were in a similar range to those for other OHCB growing on the same substrates, such as *Alcanivorax borkumensis* SK2 (Gregson *et al*., 2019) and *Thalassolituus oleivorans* MIL‐1 (Gregson *et al*., 2018).

### *Alkane‐induced proteins*

The first step in alkane degradation is the introduction of an oxygen atom to the terminal carbon of an alkane by an alkane monooxygenase producing a corresponding primary alcohol (e.g. tetradecane to 1‐tetradecanol). The genome of *O*. *antarctica* has three genes for alkane monooxygenases (C23040; C34350; C34450), which based on a census of *Gammaproteobacteria* with sequenced genomes, is the largest amount along with *Marinobacter hydrocarbonoclasticus* VT8 (Marquez and Ventosa, 2005; Kube *et al*., 2013). Other hydrocarbonoclastic bacteria are known to express only one class of alkane oxidizing enzymes (e.g. AlkB, CYP153, AlkW) (van Beilen *et al*., 2006; Lo Piccolo *et al*., 2011; Nie *et al*., 2014). However, they have multiple genes encoding different isozymes of the same enzyme which could enable the bacterium to have a broader substrate range and allow more efficient regulation of their metabolism. This may also be the case for *O*. *antarctica*.

The first alkane monooxygenase (R4YNV2/C23040), which is homologous to AlkB2 from *Alcanivorax dieselolei* B‐5 (B5T_00103) and *Alcanivorax borkumensis* SK2 (ABO_0122), was not detected in our data set. The expression of *alk* genes may be dependent on the bacterial growth phase with the expression of *alkB2* during the early exponential phase and *alkB1* in the mid to late exponential phase (Marín *et al*., 2003). As proteins were extracted in the mid‐exponential phase, this may explain why the AlkB2 homologue was not detected. In addition, it is possible that this gene would be expressed during growth on a different length hydrocarbon. There is a plenty of evidence provided by Kube *et al*. (2013) that show this gene (C23040) was acquired through HGT. C23040 is located in the region between GI GI:19 and GI:20, which is rich in transposases indicating HGT. The presence of a prophage within the genome of *O*. *antarctica* suggests phage‐mediated HGT has occurred and several GIs were also found to be homologous to the plasmid pCP301 from *Shigella* suggesting plasmid‐mediated HGT. The second alkane monooxygenase (R4YQX3/C34350) was exclusively expressed on *n*‐C_14_, whereas the third alkane monooxygenase (R4YQY4/C34450) was 12‐fold greater on *n*‐C_14_ compared with Tween indicating a small baseline constitutive expression with increased biosynthesis in response to alkane exposure. All three AlkB homologues have low sequence similarity to each other (<30%).

Upstream of both expressed alkane monooxygenases (C34350 and C34450) are open reading frames that code for proteins related to the AraC family of transcriptional regulators (C34330 and C34430) as seen in other *Alcanivorax* and *Pseudomonas* isolates. *alkB* regulation in Gram‐negative bacteria mostly belong to the AraC or LuxR family (Ratajczak *et al*., 1998; Tani *et al*., 2001; van Beilen *et al*., 2001; van Beilen *et al*., 2004; Liu *et al*., 2011). Some of the regulators in Gram‐negative bacteria can directly respond to *n*‐alkanes and induce *alkB* gene expression (Marín *et al*., 2001; Tani *et al*., 2001). These regulators are divergently transcribed with *alkB* (Whyte *et al*., 2002). The low sequence similarity between the two AraC family regulators in *O*. *antarctica* (36%) may suggest distinct regulatory mechanisms for C34350 and C34450 expression by *n*‐alkanes.

The genome also contains a P450 cytochrome (C17420) which was presumed to be involved in terminal hydroxylation of hydrocarbons (Kube *et al*., 2013). However, in all our treatments within this study, the cytochrome was not detected during growth, suggesting it is not involved in alkane degradation in *O*. *antarctica* and performs another function. This result is supported by previous work showing the CYP153 family of P450 oxygenases, which are known to be involved in alkane degradation, contain a well‐conserved N‐terminal (MFIAMDPP) and C‐terminal (HTCMGNRL) which is absent from the cytochrome P450 in *O*. *antarctica* (Kubota *et al*., 2005; Wang *et al*., 2010).

The second and third step in alkane degradation is the conversion of the primary alcohol to its corresponding aldehyde catalysed by an alcohol dehydrogenase, followed by conversion of the aldehyde to its corresponding fatty acid by an aldehyde dehydrogenase. The alcohol dehydrogenase AlkJ (R4YRA7/C34360) shows amino acid homology to AlkJ from *Alcanivorax borkumensis* SK2 (van Beilen *et al*., 1992). *Oleispira antarctica* also expressed an oxidoreductase (R4YJ92/C00500) that is homologous to an alcohol dehydrogenase LaoA from *Pseudomonas aeruginosa* (PA0364; 58% identity). LaoA from *P*. *aeruginosa* is part of a gene cluster that was shown to oxidize the alcohols derived from the alkane degradation and also contains an inner membrane transport protein (LaoB/PA0365), an aldehyde dehydrogenase (LaoC/PA0366) and a transcriptional regulator (LaoR/PA0367) (Panasia and Philipp, 2018). This genetic organization is also seen in *O*. *antarctica* as the oxidoreductase (C00500) which is part of a cluster consisting of genes coding for an inner membrane transport protein (C00510), an aldehyde dehydrogenase (C00520) and a TetR transcriptional regulator (C00530). Given that AlkJ (C34360) shows low similarity (31% identity) to LaoA (C00500), this means there are two distinct alcohol dehydrogenase mechanisms both active under alkane‐degrading conditions in *O*. *antarctica*. In addition, we checked for this genetic organization (alcohol dehydrogenase, inner membrane transport protein, aldehyde dehydrogenase and transcriptional regulator grouped in an operon) in the genomes of other key marine obligate *n*‐alkane‐degrading bacteria and found the same organization in *Alcanivorax borkumensis* SK2 (ABO_0085–ABO_0088; Schneiker *et al*., 2006), *Alcanivorax dieselolei* B5 (B5T_00037–B5T_00040; Lai *et al*., 2012), *Alcanivorax jadensis* T9A (T9A_01032–T9A_01035; Parks *et al*., 2017), *Thalassolituus oleivorans* MIL‐1 (TOL_0221–TOL_0224; Golyshin *et al*., 2013) and *Marinobacter hydrocarbonoclasticus* SP17 (MARHY_3473–MARHY_3476; Grimaud *et al*., 2012). The increased protein biosynthesis of this system observed in our analysis of *O*. *antarctica* and observations in the genomes other OHCB suggest this enzyme system is not just restricted to *P*. *aeruginosa* and may be much more widespread among other marine hydrocarbon‐degrading bacteria. This may represent an additional/alternative oxidation system for the alcohols derived from alkane degradation compared with systems involving the well characterized dehydrogenase AlkJ, potentially enhancing the substrate range of these marine hydrocarbon‐degrading bacteria.

### *Cold‐induced proteins*

As bacterial cells move through heterogenous environments, they encounter various fluids of different viscosities. Under changing conditions (e.g. temperature), bacteria experience different levels of viscous drag (or mechanical load) on their flagella. The physical properties of water are temperature‐dependent, particularly viscosity. A change in temperature from 16°C to 4°C is associated with an increase in kinematic viscosity from 1.0508 × 10^−6^ to 1.6262 × 10^−6^ m^2^ s^−1^. The evidence that *O*. *antarctica* is responding to a higher viscosity environment at 4°C was the increased biosynthesis of particular aspects of the flagellar that will generate sufficient force for rotation including FleQ, FliL and FlaB. Flagella assembly is controlled by FleQ, an NtrC‐like transcriptional regulator which controls synthesis of flagellar genes and its expression, in other flagellated bacteria, was shown to be cold‐induced, based on an 83% decrease in *fleQ* transcripts at 30°C compared with 4°C (Soutourina *et al*., 2001). The flagellar protein, FliL, which is a single‐transmembrane protein with a large periplasmic region and associates with the flagellar basal body, has been found to be important for the function of the motor under high‐load conditions, such as highly viscous environments, by recruiting or stabilizing the stators or by increasing their efficiency, leading to greater torque generation (Partridge *et al*., 2015; Takekawa *et al*., 2019). Flagellin is the major structural protein of the flagella filaments, which in *O*. *antarctica* is composed of the subunits FlaA and FlaB. It has been suggested that by altering the ratio of the flagellin subunits the shape of the filament may be optimally adapted to different environmental circumstances (e.g. greater density), with FlaB protein levels increasing at lower temperatures (Wösten *et al*., 2010). The relative amounts of the flagellin subunits could determine the degree of flexibility or stiffness of the flagellum, and the modulation of these amounts, with increased FlaB expression, adapts the organelle to changes in the viscosity experienced by *O*. *antarctica*.

The increased viscosity of fluids at lower temperatures will also influence the motility of bacteria travelling through them. *Oleispira antarctica* has a monopolar, monotrichous flagellum meaning it lacks the ‘run‐and‐tumble’ motility mechanism seen in the peritrichous *E*. *coli* (Kube *et al*., 2013). It most likely uses ‘run‐and‐reverse’ swimming where the flagellum rotates counterclockwise (CCW) to push the cell forward and clockwise (CW) to pull it backwards. This type of motility is very prevalent in the ocean being used in approximately 70% of marine isolates (Johansen *et al*., 2002). The main cause to increase the flagella switching frequency (CCW to CW) is the chemotaxis signalling pathway, and several components of this pathway showed increased biosynthesis at 4°C. In the pathway, stimulation of the chemotaxis receptors, also known as MCPs, is followed by phosphorylation or acetylation of the response regulator CheY; phosphorylated (CheY‐P) or acetylated (CheY‐Asc); CheY then associates with the rotor of the flagella motor, and the motor changes direction to CW (Porter *et al*., 2011; Takabe *et al*., 2017). Six MCPs showed increased biosynthesis (Fig. 3B) and may be detecting changes in temperature (thermoreceptors), viscosity (viscoreceptors), or both, through their periplasmic domains. Following activation and signal transduction through these MCPs, *O*. *antarctica* needs to adapt to a background level of the attractant (i.e. temperature/viscosity) by resetting the receptor proteins to a non‐signalling state. This is done by CheR that was exclusively expressed at 4°C (Fig. 3B), which methylates conserved glutamate residues in the cytoplasmic signalling domain of the MCPs. The response regulators, CheY, in *O*. *antarctica*, are most likely activated through acetylation rather than phosphorylation due to the 12‐fold increased biosynthesis of acetyl‐CoA synthetase (AcsA), which adds an acetyl group at conserved lysine residues (Barak and Eisenbach, 2001). This is also reinforced by the increased biosynthesis of CheZ which dephosphorylates CheY‐P (Barak and Eisenbach, 2004). The increased biosynthesis of these proteins indicates a shift to CW flagellar rotation which would lead to more reversal events. *Oleispira antarctica* may be a poor swimmer in low viscosity environments, and this could be one of the reasons it is outcompeted in more temperate areas. The viscosity‐induced increase in reversal events would limit cell migration, resulting in the accumulation of *O*. *antarctica* cells in an area where it can outcompete other bacteria. Alternatively, the cells could be more stressed at 16°C, as it is slightly outside the growth optima of *O*. *antarctica*, and are looking for a better environment (colder) to accumulate.

Low temperatures also increase the production of reactive oxygen species (ROS) due to the generation of heat accompanied by increased respiration and therefore oxygen consumption. ROS induce different forms of cell damage, disturb the redox state and can change the activity of several metabolic enzymes leading to oxidative stress (Blagojevic *et al*., 2011). Evidence to suggest *O*. *antarctica* is counteracting oxidative stress at lower temperatures was the increased biosynthesis of the proline utilization protein, PutA, and the catalase‐peroxidase, KatG. Previous studies have suggested that proline metabolism increases oxidative stress resistance (Zhang *et al*., 2015). PutA catalyses the oxidation of proline to glutamate and is made up of two domains, a proline dehydrogenase (PRODH) domain, which couples the two electron oxidation of proline with the reduction of ubiquinone, and a delta‐1‐prroline‐5‐carboxylate dehydrogenase (P5CDH) domain, that converts a metabolic intermediate (P5C) into glutamate generating NADH (Menzel and Roth, 1981; Moxley *et al*., 2011). Proline metabolism will generate hydrogen peroxide (H_2_O_2_) through the PRODH domain with electrons going into the ubiquinone pool and the P5CDH domain produces NADH, both of which would lead to increased electron flux through the respiratory chain. Increases in the endogenous levels of H_2_O_2_ would then be enough to induce expression of proteins active against ROS e.g. KatG. Hydroperoxidase I (coded by *katG*) expression is induced by H_2_O_2_ and when expressed is active against ROS (Zhang *et al*., 2015). This indicates proline metabolism via PutA may offer *O*. *antarctica* a competitive advantage over other bacteria in harsh oxidative/low temperature environments through a preadaptive effect involving greater endogenous H_2_O_2_ production and enhanced peroxidase expression.

*Oleispira antarctica* has previously been shown to express cold‐adapted chaperonins Cpn60 (C29610) and Cpn10 (C29620) which are homologous to the *E*. *coli* GroELS system, promoting the folding and assembly of over 30% of *E*. *coli*'s cellular proteins (Gething and Sambrook, 1992; Ferrer *et al*., 2003; Kube *et al*., 2013). GroELS rapidly loses its refolding activity at temperatures below 37°C but Cpn60/Cpn10 functions well at low temperatures (Ferrer *et al*., 2003). Coexpression of Cpn60/Cpn10 from *O*. *antarctica* in *E*. *coli* lowered its minimal growth temperature below 15°C (Ferrer *et al*., 2003, 2004). These findings indicated the chaperonins play a key role in cold sensitivity, adaption or tolerance. However, Cpn60 was abundantly detected at both the warmer 16°C and colder 4°C temperature, suggesting these chaperonins are required for protein folding at all temperatures (Table S2). *Oleispira antarctica* also has a GroELS chaperonin system coded by its genome. The biosynthesis of GroEL (C29610) chaperonin was 1.6‐fold higher at 16°C and the GroES (C29620) chaperonin was not detected at either temperature, indicating this system is induced by and folds protein at higher temperatures.

## Conclusion

This study contributes to our understanding of a key psychrophilic hydrocarbon‐degrading bacterium that dominates microbial communities in cold marine environments following oil spills. It has demonstrated that *O*. *antarctica* has a very restricted substrate range, even compared with other OHCB, and might explain why it is outcompeted in warmer oil‐contaminated marine environments by more metabolically versatile hydrocarbon degraders. The study also identified key hydrocarbon‐degrading enzymes relating their expression to the physiological activity of *O*. *antarctica* and validated previous functional genomic expression analysis performed in Kube *et al*. (2013) that putatively assigned genes to alkane degradation pathways. This study also highlights potential mechanisms *O*. *antarctica* utilizes that may provide it with ecological competitiveness at low temperatures. These include structural changes in the flagella to generate sufficient force to counteract increased viscosity, use of chemotaxis machinery to alter flagella rotation causing accumulation of cells in beneficial areas and increased proline metabolism to generate H_2_O_2_ enhancing hydroperoxidase expression to counteract oxidative stress. Overall, we have a much deeper insight into the life of an OHCB in the cold, but whether these metabolic pathways and adaptation mechanisms exist in other marine hydrocarbon‐degrading psychrophiles requires further investigation.

## Experimental procedures

### Culture conditions and growth of O. antarctica RB‐8

Cultures of *O*. *antarctica* RB‐8 (DSM 14852) were established in sterile 100 ml culture flasks containing 50 ml of ONR7a media (Dyksterhouse *et al*., 1995). Triplicate cultures were established for each temperature and time point to be sampled that were enriched separately with the following alkanes at a final concentration of 0.1% w/v: decane (C_10_), dodecane (C_12_), hexadecane (C_16_), eicosane (C_20_), tetracosane (C_24_), octacosane (C_28_) and dotriacontane (C_32_) and the branched alkane pristine (Sigma‐Aldrich). Non‐hydrocarbon controls cultures were established with Tween 80 (polyethylene glycol sorbitan mono‐oleate) at 1% v/v. Tween 80 was chosen to be the non‐hydrocarbon control as OHCB exhibit a ‘BIOLOG anomaly’ i. e. growth occurs on only 2 of the 95 organic growth substrates in the BIOLOG1 system, namely Tween 40 and Tween 80, substrates that contain long‐chain alkyl moieties. No‐carbon controls (ONR7a media with no added carbon source) were also established along with uninoculated cultures to determine whether any hydrocarbon losses were abiotic. The cultures were incubated at 4°C and 16°C, at 60 rpm, for 21 days. Growth curves were determined from increase in optical density over time at 600 nm on a NanoDrop 1000 Spectrophotometer.

### *GC–MS analysis*

Alkanes were extracted in 5 ml of hexane or hexane:dichloromethane (1:1) for C_32_. The vials were shaken vigorously. Samples were then centrifuged (4600*g*, 15 min). One millilitre of the upper solvent phase was taken and diluted with hexane to an appropriate concentration for GC–MS analysis. Deuterated nonadecane (C_19_^d40^) was added as an internal standard at 5 μg ml^−1^. Alkanes were identified and quantified using a TRACE Ultra Gas Chromatograph (ThermoFisher Scientific) coupled with a TRACE DSQ Mass Spectrometer (ThermoFisher Scientific) operated at 70 eV in positive ion mode. Chromatography was performed by splitless injection with helium as the carrier gas, onto a 30 m × 0.25 mm × 0.25 mm fused silica capillary column Rtx‐5MS (Restek) (0.25 μm film thickness). The injector temperature was 300°C, and the oven program was 65°C for 2 min, increasing to 310°C at 20°C min^−1^ then held for 18 min. External multilevel calibrations were performed using an alkane standard mix (C_8_‐C_40_) (Sigma‐Aldrich) with quantification of five levels ranging from 0.250–16 ng μl^−1^. The mass spectrometer was operated in full scan mode (range *m/z* 50–650), with identification of target analytes based on retention times of the analytical standards and mass spectrum (intensity versus *m/z*). Quantification was performed by integrating the peak of target analytes at specific *m/z* ratios.

#### *Proteomic analysis*

Cultures of *O*. *antarctica* RB‐8 (DSM No: 14852, Type strain) were grown in 160 ml culture flasks containing 50 ml of ONR7a artificial seawater media (Dyksterhouse *et al*., 1995 supplemented with either *n*‐tetradecane (*n‐*C_14_) (Sigma‐Aldrich) or the non‐hydrocarbon control Tween 80 (0.1% v/v) as the sole carbon source and incubated at either 4°C or 16°C (*n* = 3). Cells were harvested for protein extraction after 4 days in mid‐exponential phase by centrifugation (4600*g*, 15 min). The cell pellet was washed in 2 ml of Dulbecco's PBS (Sigma‐Aldrich) centrifuged (10,500*g*, 5 min). Total proteins was extracted by resuspending the cell pellet in 50 μl of protein extraction buffer (62.5 mM TRIS, 10% glycerol w/v, 12 mM dithiothreitol, 2% SDS v/v and 1 Pierce Protease Inhibitor Tablet per 50 ml) and heating in a water bath (95°C, 12 min) fully lysing the cells, and then centrifuged (10,500*g*, 5 min) to remove cell debris. Proteins extracts were visualized by SDS‐PAGE before performing in‐gel digestion with trypsin and subsequent analysis of the peptides on a hybrid high‐resolution LTQ/Orbitrap Velos LC–MS/MS instrument (Thermo Scientific) as previously described (McKew *et al*., 2013).

#### *MS/MS analysis*

MS/MS analysis was performed using the methods previously used in Gregson *et al*., 2018. Uniprot protein sequences from the *O*. *antarctica* RB‐8 genome (Kube *et al*., 2013) were used to perform identification. Proteins were validated using the default settings in MaxQuant and Andromeda with a minimum of one peptide, but that any such protein had to be unambiguously identified by peptides that were unique to that protein. Spectral counts were normalized using the Total Spectral Count (TSpC) method (Dong *et al*., 2007) where the sample with the highest number of TSpC is chosen and the remaining samples are normalized to it to account for small differences in total detected spectral counts per run. The Normalized Spectral Abundance Factor (number of spectral counts divided by the length of the polypeptide, expressed as percentage for each protein compared with the sum of this ratio for all the detected proteins) was also calculated as longer proteins are expected to produce more peptides (Florens *et al*., 2006; Zybailov *et al*., 2006).

#### *Statistical analysis*

Differential expression analysis was performed on normalized spectral count data in the OMICS package of XLSTAT‐Premium Version 2016.1 (Addinsoft) to identify differentially expressed proteins by analysis of variance with Tukey Honestly Significant Difference post hoc test for pairwise comparisons, according to the factors ‘substrate’ (two levels: *n‐*C_14_, Tween 80) and ‘temperature’ (two levels: 4°C or 16°C). The Benjamini‐Hochberg False Discovery Rate corrections procedure was used for post hoc *P* value corrections (Benjamini and Hochberg, 1995).

### *Bioinformatic analysis*

All proteins showing a significant (*P* < 0.05) increase in spectral count on hydrocarbons were subjected to a BLASTp (Basic Local Alignment Search Tool) (Altschul *et al*., 1990) homology search where the protein was compared with the nr database (non‐redundant sequences from GenBank CDS translations, PDB, Swiss‐Prot, PIR and PRF). Protein family and domain analysis was carried out in Pfam v30.0 (Finn *et al*., 2016). SCOOP (Simple Comparison of Outputs Program) (Bateman and Finn, 2007) was used to detect relationships between families in the Pfam database. Proteins were assigned to functional families by hierarchical classification of protein domains based on their folding patterns in CATH v4.1 (Class, Architecture, Topology, Homology) (Sillitoe *et al*., 2015). Full length secondary and tertiary structure predictions, functional annotations on ligand‐binding sites, enzyme commission numbers and gene ontology terms were generated using the I‐TASSER SERVER (Zhang, 2008).

## Supporting information


**Fig. S1** ‐ Growth of *Oleispira antarctica* RB‐8 at 4°C (left) and 16°C (right) in ONR7a media enriched with aliphatic *n*‐alkanes (*n*‐C_10_‐decane, *n*‐C_12_‐dodecane, *n*‐C_16_‐hexadecane, *n*‐C_20_‐eicosane, *n*‐C_24_‐tetracosane) and a non‐hydrocarbon control (Tween 80) at 0.1% and 1% (w/v) respectively (means ± SE; *n* = 3).

**Fig. S2** ‐ Degradation (%) of aliphatic *n*‐alkanes (n‐C10‐decane; n‐C12‐dodecane; n‐C16‐hexadecane; n‐C20‐eicosane; n‐C24‐tetracosane) at 4°C and 16°C after 21 days in ONR7a media inoculated with *Oleispira antarctica* RB‐8 (means ± SE; *n* = 3).

**Table S1** ‐ Growth Rates of *Oleispira antarctica* RB‐8 at 4°C (left) and 16°C in ONR7a media enriched with aliphatic n‐alkanes (n‐C10‐decane, n‐C12‐dodecane, n‐C16‐hexadecane, n‐C20‐eicosane, n‐C24‐tetracosane).

**Table S2** Mean spectral counts (SpC; n = 3) of expressed proteins during growth on a medium‐chain alkane (n‐C14) and a non‐hydrocarbon control (Tween) in *Oleispira antarctica* RB‐8 at two different temperatures (4°C and 16°C), with differential expression analysis (Anova with Benjamini‐Hochberg FDR correction (P‐value) and Tukey HSD Post Hoc Test (a, b, c in parentheses indicate significant differences by Tukey HSD test)).
