# Supplementary material for: Protein expression in the obligate hydrocarbon‐degrading psychrophile Oleispira antarctica RB‐8 during alkane degradation and cold tolerance
Source: Environ Microbiol. 2020 Feb 28;22(5):1870–83. doi: 10.1111/1462-2920.14956 (PMC7318663; doi:10.1111/1462-2920.14956)
Supplement: Supplementary file 1 — Fig. S1 ‐ Growth of Oleispira antarctica RB‐8 at 4°C (left) and 16°C (right) in ONR7a media enriched with aliphatic n‐alkanes (n‐C10‐decane, n‐C12‐dodecane, n‐C16‐hexadecane, n‐C20‐eicosane, n‐C24‐tetracosane) and a non‐hydrocarbon control (Tween 80) at 0.1% and 1% (w/v) respectively (means ± SE; n = 3). [file EMI-22-1870-s001.pdf]

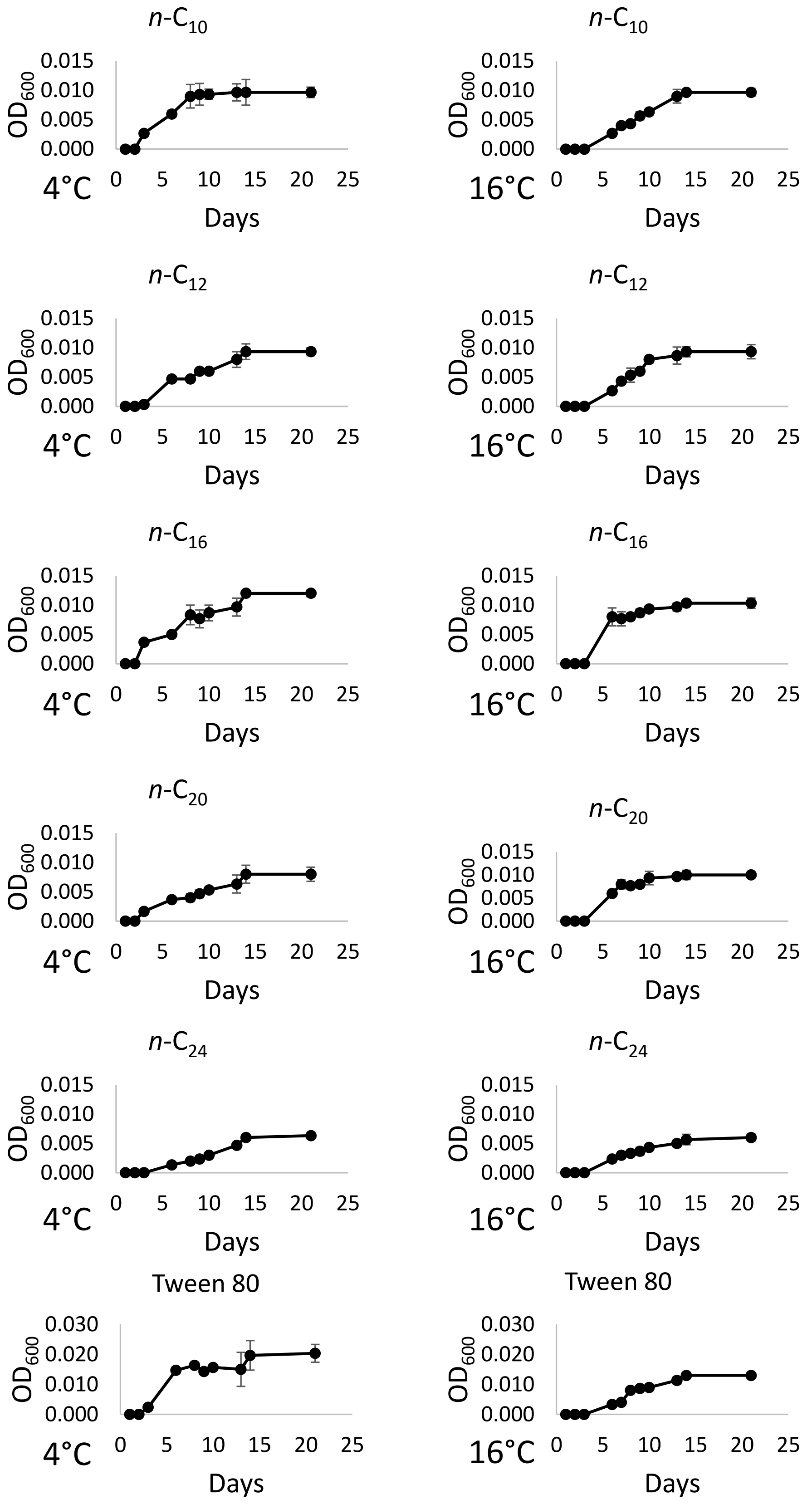

Fig S1 - Growth of *Oleispira antarctica* RB-8 at 4°C (left) and 16°C (right) in ONR7a media enriched with aliphatic *n*-alkanes (*n*-C_{10}-decane, *n*-C_{12}-dodecane, *n*-C_{16}-hexadecane, *n*-C_{20}-eicosane, *n*-C_{24}-tetracosane) and a non-hydrocarbon control (Tween 80) at 0.1% and 1% (w/v), respectively (means ± SE; *n*=3)
