# Supplementary material for: Protein expression in the obligate hydrocarbon‐degrading psychrophile Oleispira antarctica RB‐8 during alkane degradation and cold tolerance
Source: Environ Microbiol. 2020 Feb 28;22(5):1870–83. doi: 10.1111/1462-2920.14956 (PMC7318663; doi:10.1111/1462-2920.14956)
Supplement: Supplementary file 2 — Fig. S2 ‐ Degradation (%) of aliphatic n‐alkanes (n‐C10‐decane; n‐C12‐dodecane; n‐C16‐hexadecane; n‐C20‐eicosane; n‐C24‐tetracosane) at 4°C and 16°C after 21 days in ONR7a media inoculated with Oleispira antarctica RB‐8 (means ± SE; n = 3). [file EMI-22-1870-s002.pdf]

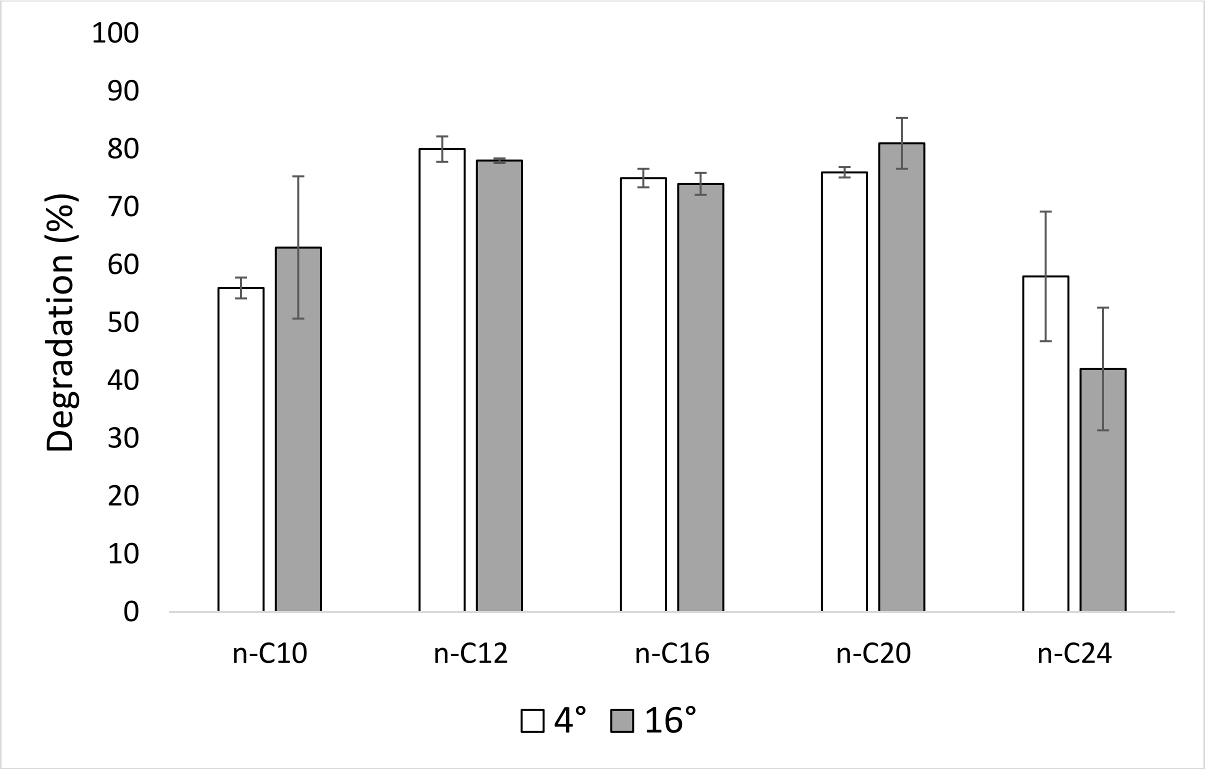

Fig S2 – Degradation (%) of aliphatic *n*-alkanes (n-C10-decane; n-C12-dodecane; n-C16-hexadecane; n-C20-eicosane; n-C24-tetracosane) at 4°C and 16°C after 21 days in ONR7a media inoculated with *Oleispira antarctica* RB-8 (means ± SE; *n*=3)
